# Aggressive hydration with lactated ringer solution in prevention of post-endoscopic retrograde cholangiopancreatography pancreatitis: A systematic review and meta-analysis

**DOI:** 10.1097/MD.0000000000025598

**Published:** 2021-04-23

**Authors:** Mengmeng Wu, Shuaiyu Jiang, Xiaoguang Lu, Yilong Zhong, Yi Song, Zhiwei Fan, Xin Kang

**Affiliations:** a Graduate School, Dalian University, Dalian; b Department of Emergency Medicine, Zhongshan Hospital, Dalian University, Dalian, China.

**Keywords:** endoscopic retrograde cholangiopancreatography, Lactated Ringer Solution, Meta-analysis, pancreatitis, Systematic review

## Abstract

**Background:**

Acute pancreatitis is the most common complication of Endoscopic Retrograde Cholangiopancreatography (ERCP). There was no conclusion on the prevention of Post-ERCP Pancreatitis (PEP) by Lactated Ringer Solution.

**Aim:**

The purpose of this meta analyses is to determine whether aggressive hydration with Lactated Ringer Solution reduced the incidence of PEP.

**Methods:**

We retrieved randomized clinical trials comparing the preventive effects of aggressive hydration with Lactated Ringer Solution and standard hydration on PEP from PubMed, the Cochrane Library, Embase, the Web of Science, Clinical Trial.gov, Scopus database, CNKI, CQVIP and WanFang Data. Primary outcome was incidence of PEP. Secondary outcomes included incidence of hyperamylasemia, abdominal pain and adverse events.

**Results:**

Ten randomized controlled trials with 2200 patients were included in this meta-analysis. Meta-analysis showed that compared with standard hydration, aggressive hydration reduced the incidence of PEP (odds ratio [OR], 0.40; 95% confidence intervals [CI], 0.26–0.63; *P* < .0001). Compared with standard hydration, aggressive hydration also reduced the incidence of hyperamylasemia after ERCP (OR, 0.48; 95% CI, 0.38–0.60; *P* < .0001). There was significant difference between aggressive hydration and standard hydration in the incidence of abdominal pain (OR, 0.29; 95% CI, 0.11–0.73; *P* = .008). There was no difference in adverse events between aggressive hydration and standard hydration (OR, 0.93; 95% CI, 0.21–4.13; *P* = .93). Sensitivity analyses showed that neither alternative effect measures nor statistical models regarding heterogeneity affected the conclusions of this meta-analysis.

**Conclusion:**

Aggressive hydration with Lactated Ringer Solution during perioperative period of ERCP can prevent PEP.

## 1 Introduction

Endoscopic retrograde cholangiopancreatography (ERCP) is an important diagnostic and therapeutic method for pancreatic and biliary diseases. With the development of endoscopic technology, ERCP has been widely used in clinic and has become a standard method for endoscopic minimally invasive diagnosis and treatment of biliary and pancreatic diseases.^[1]^ Although ERCP is considered safe, it is one of operations that causes the most complications in endoscopic surgery.^[2]^ Post-Endoscopic Retrograde Cholangiopancreatography Pancreatitis (PEP) is the most common complication of ERCP. ERCP refers to the insertion of endoscopy into the lower part of the duodenum and the injection of contrast agent into the pancreaticobiliary duct through the opening of the duodenal papilla or fistula. There are several potential mechanisms of pancreatic injury during ERCP, including mechanical, thermal, chemical, hydrostatic, enzymatic and microbial damage.^[3]^ Long-term operation of papillary foramen, cannulation of biliary tree and repeated cannulation lead to duct injury or ampullary injury.^[4]^ Thermal injury can be caused by electric knife current used in sphincterotomy (biliary or pancreatic), endoscopic papillectomy or ablation of tumors in Vater ampulla.^[5]^ Papillary edema caused by mechanical or thermal injury is thought to hinder the outflow of pancreatic secretions, leading to pancreatitis. Contrast agents may cause PEP by causing chemical damage; however, the data are still controversial.^[6]^

PEP is defined as pancreatitis-related clinical symptoms lasting more than 24 hours after ERCP, accompanied by serum amylase three times higher than the upper limit of normal value.^[7]^ It is called hyperamylasemia after ERCP if only amylase is elevated after ERCP without no clinical manifestation of pancreatitis. There are many factors that increase the risk of PEP including patient-related factors, endoscopist-related factors and procedure-related factors.^[8–10]^ Therefore, chemical prevention and procedural techniques for PEP prevention should take all these factors into account.^[11]^ Most of the complications are mild to moderate, a few are severe, and even can lead to death. Therefore, how to prevent PEP is a hot topic in clinical research. The PEP risk of low-risk and high-risk patients was higher than 5% and 16%, respectively.^[12]^ To minimize this risk, clinicians must select the right patients, use ERCP almost exclusively as a treatment procedure, use reasonable procedural techniques, and consider the use of rectal indomethacin, active hydration, or placement of pancreatic stents.^[13]^ Large randomized controlled trials need to further determine which patient groups benefit from rectal indomethacin treatment and determine the role of pancreatic duct stenting in low-risk patients.

There are many studies on preventive drugs for PEP, but the conclusions are inconsistent, and there is no unified PEP drug prevention standard in clinical practice.^[14]^ The best PEP prophylactic drugs should have the characteristics of effective, less side effects, high cost-benefit ratio, easy access and convenient administration. Although there are many clinical reports, only a few have definite preventive value. Non-steroidal anti-inflammatory drugs and guidewire-assisted cannulation technique are currently recommended as prevention for PEP.^[15,16]^ But according to a systematic review, the protection of pancreatic duct stent implantation may be limited to patients with moderate but non-severe PEP.^[13]^ In recent years, infusion therapy for pancreatitis has attracted more and more attention. Fluid therapy is an important part of early treatment of acute pancreatitis. Fluid therapy reduces hypovolemic shock, which is usually associated with acute pancreatitis, improves pancreatic microvascular perfusion, and thus improves the prognosis of patients.^[17]^ However the results of a national survey conducted in 2009 by the Pancreatic Disease Research Council supported by MHLW in Japan showed that all patients under 60 who died of severe pancreatitis had insufficient infusion volume (less than 50 ml/kg) within 24 hours of starting infusion therapy. Compared with healthy adults who need 1500 to 2000 ml of water per day (25–30 ml/kg body weight), patients with early acute pancreatitis need 2–5 times the infusion volume (50–150 ml/kg body weight).^[18]^ We define the former as standard hydration and the latter as aggressive hydration in this article. Moreover, in clinical acute pancreatitis, the optimal time, volume and effects of fluid therapy are uncertain. In view of some contraindications, the preventive effect of Lactated Ringer Solution on PEP needs to be studied. There were three previous meta-analyses on this topic,^[19,20]^ but the sample size of meta-analysis was relatively small. Therefore, we conducted an updated study on the previous meta-analysis.

## 2 Materials and methods

### 2.1 Study selection and data collection

The present study conducted a comprehensive literature search of PubMed, the Cochrane Library, Embase, the Web of Science, Scopus database, CNKI, CQVIP and WanFang Data to identify the relevant articles published before April 2019. Database search was conducted with the combination of the following searching terms: (“endoscopic retrograde cholangiopancreatography” or “ERCP” or “post-ERCP pancreatitis” or “cholangiopancreatography” or “pancreatitis” or “PE” or “pancreas” or “pancreatic” or “pancreatic disease”)AND (“aggressive hydration” or “vigorous hydration” or “hydration” or ”infusion” or “solution” or “fluid resuscitation” or “Ringer solution” or “Ringer's solution” or “lactated solution” or “Lactated Ringer's solution“ or Lactated Ringer solution” or “Lactated Ringer's”) and the combined phrases. Reference lists of each article, relevant meta-analyses and reviews were also searched.

### 2.2 Inclusion and exclusion criteria

#### 2.2.1 Inclusion criteria

1. The definition of PEP was defined according to consensus criteria as follows: serum amylase levels were 3 times higher than the upper normal limit at 24 hours after surgery and the presence of continuous pancreatitis-like abdominal pain.^[7]^
2. randomized controlled trials (RCTs) conducted in adults who received ERCP procedures for any reason.
3. RCTs comparing aggressive hydration with Ringer's lactate solution with standard hydration on PEP prophylaxis.
4. The primary endpoint was defined as the incidence of PEP. The secondary endpoints were defined as the incidence of hyperamylasemia, the incidence of adverse effect.

#### 2.2.2 Exclusion criteria

1. Duplicate publications
2. Combinations of other pancreatitis prevention methods (Non-steroidal anti-inflammatory drugs and pancreatic duct stent).
3. Past ERCP history.
4. Nonrandomized studies.

#### 2.2.3 Data Extraction and Quality Assessment

Two authors screened the flow diagram and completed the data extraction. The data extraction items included: primary author, publication year, sample size, intervention, PEP diagnostic criteria, PEP rate, hyperamylasemia rate, and adverse effect rate in each study. The methodological quality of the relevant studies was assessed according to the recommendations of Cochrane Handbook for Systematic Reviews of Interventions. To evaluate this meta-analysis comprehensively and guide clinical practice, we assessed the quality of evidence with the GRADE pro software. The following domains were considered to assess the risk of bias in this meta-analysis: the sequence generation, allocation concealment, blinding of participants and personnel, blinding of outcome assessment, incomplete outcome data, selective reporting and other bias. Two authors (M.-M.W. and X.-G.L.) evaluated the quality of evidence according to the GRADE quality assessment criteria. Disagreements were resolved by discussion or presentation to the third researcher for consultation. The quality of evidence falls into four categories (high, moderate, low, or very low).

### 2.3 Statistical analysis

We used Review Manager 5.3 and Stata 12.0 software for the meta-analysis. We computed a pooled odds ratio (OR) and 95% confidence intervals (CI) by using to generate forest plots, to determine whether there was a statistical association between infusion and PEP and to assess heterogeneity of studies. Heterogeneity was quantified evaluated using the chi-square based Cochran *Q* statistic and the *I*^2^ statistic, this statistic yields results ranging from 0 to 100% (*I*^2^ = 0–25%, no heterogeneity; *I*^2^ = 25–50%, moderate heterogeneity; *I*^2^ = 50% to 75%, large heterogeneity; and *I*^2^ = 75% to 100%, extreme heterogeneity). If heterogeneity existed, we used the random effects model, otherwise, we used the fixed effects model. In addition, we analyzed which factors influence heterogeneity. Sensitivity analyses were used to test the robustness of the overall analysis. We assessed publication bias by inspecting a funnel plot visually. We conducted subgroup analyses when heterogeneity was present.

### 2.4 Ethical approval

No ethical approval is necessary in this study, because this article does not contain any studies with human participants or animals performed by any of the authors.

## 3 Results

### 3.1 Search results

**Figure 1:**
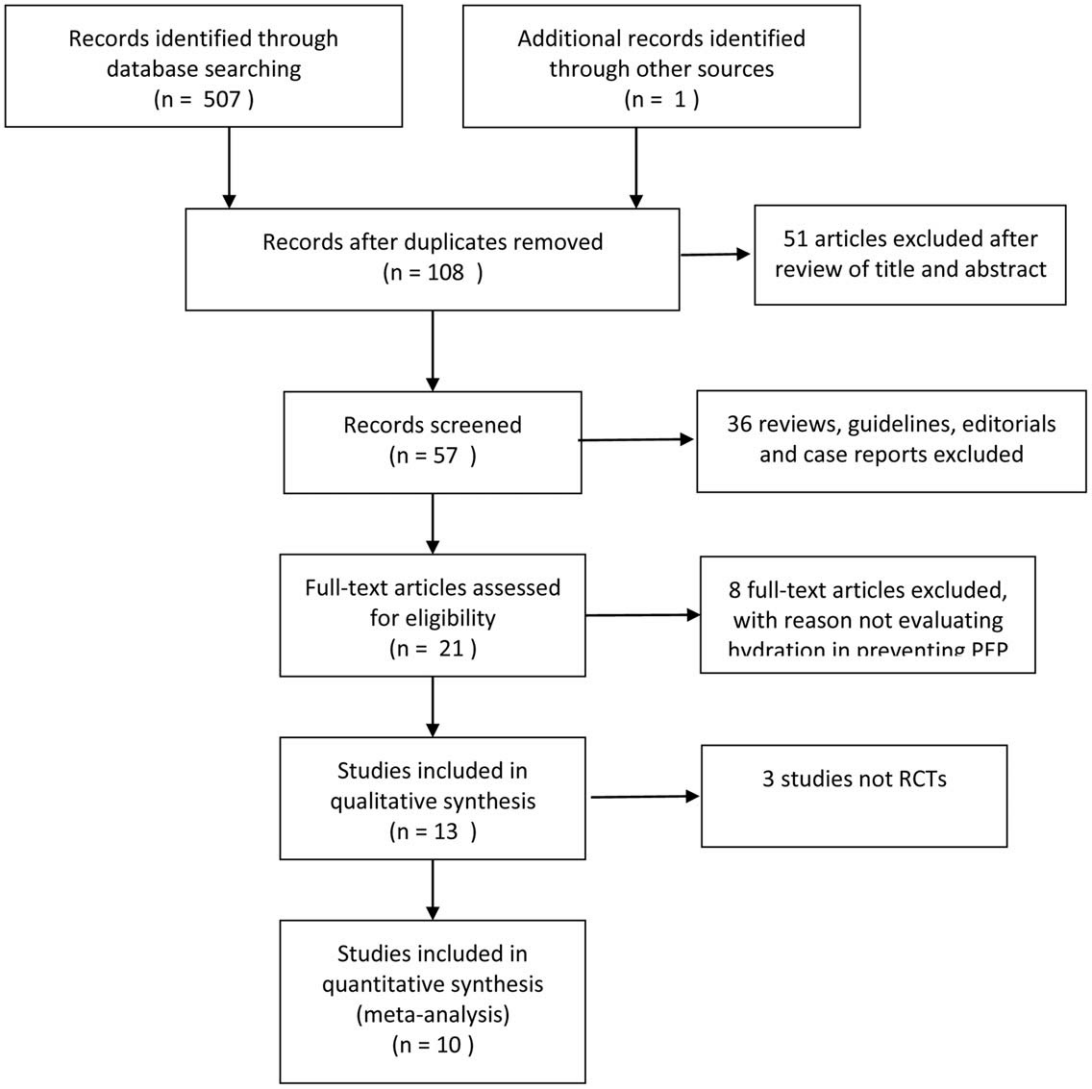
The PRISMA flow diagram of selected studies.

A total of 507 articles were found from PubMed, the Cochrane Library, Embase, the Web of Science, Scopus database, CNKI and WanFang Data. The data flow chart of document retrieval is shown in Figure 1.

### 3.2 Characteristics of trials included

Ten randomized controlled trials^[21–30]^ were included in this meta analyses. Of the 10 RCTs, two studies were in USA,^[21,22]^ two were in Korea,^[23,24]^ one study was in Ecuador^[25]^ two studies were in Iran,^[26,27]^ one study was in Portugal,^[28]^ and two studies were in Thailand.^[29,30]^ The 5 of 10 RCTs were published as full text articles, 4 RCTs were published as abstract, and one study^[21]^ was available at: https://clinicaltrials.gov/show/NCT02050048 Accessed June 26, 2016. There was no significant difference between the experimental group and the control group in procedural time, trials of cannulation, causes of obstruction, single or many operators in each study, unintentional cannulation or contrasting of the pancreatic duct. More details were shown in Table 1.

**Table 1:** Characteristics of included studies.

References	Location	Sample Size (AH/SH)	Intervention (AH/SH)	Perioperative fluid infusion dose (patient weighing 75 kg)
Buxbaum et al	USA	39/23	AH: intravenous lactated Ringer solution at a rate of 3.0mL/kg/h during the procedure, a bolus of 20mL/kg immediately after ERCP, followed by a post-ERCP rate of 3.0mL/kg/h for 8 h	AH: 3525 mL
			SH: intravenous lactated Ringer solution at a rate of 1.5mL/kg/h during ERCP and for 8h after ERCP without a bolus	SH: 1012.5 mL
Shaygan-nejad et al	Iran	75/75	AH: intravenous lactated Ringer solution at a rate of 3.0mL/kg/h during ERCP, a bolus of 20mL/kg right after ERCP and 3.0mL/kg/h of lactated Ringer solution for 8h	AH: 3525 mL
			SH: intravenous lactated Ringer solution at a rate of 1.5mL/kg/h during ERCP and the following 8h	SH: 1012.5 mL
Chuankrerkkul et al	Thailand	30/30	AH: intravenous lactated Ringer solution at a rate of 3.0mL/kg/h during ERCP, 10mL/kg bolus, and 3.0mL/kg/h for 8 h after ERCP	AH: 2775 mL
			SH: intravenous lactated Ringer solution at a rate of 1.5mL/kg/h during ERCP and 8h after ERCP	SH: 1012.5mL
Rosa et al	Portugal	35/33	AH: intravenous lactated Ringer solution at a rate of 3.0mL/kg/h during ERCP, 20mL/kg bolus after ERCP, and 3mL/kg/h for 8h after ERCP	AH: 3525 mL
			SH: intravenous lactated Ringer solution at a rate of 1.5mL/kg/h during and for 8h after ERCP	SH: 1012.5 mL
Chang et al	Thailand	85/86	AH: intravenous lactated Ringer solution at a rate of 150mL/h starting 2 h before ERCP, and continued during and after ERCP to complete 24 h	AH: 3600 mL
			SH: intravenous lactated Ringer solution calculated by the Holliday-Segar method given peri-ERCP	SH: Not described
Choi et al	Korea	255/255	AH: lactated Ringer solution in an initial bolus of 10mL/kg before ERCP, 3.0mL/kg/h during and for 8h after ERCP, and a post-ERCP bolus of 10mL/kg	AH: 2775 mL
			SH: lactated Ringer solution at a rate of 1.5mL/kg/ h during and for 8h after ERCP	SH: 1012.5mL
NCT02050048	USA	14/12	AH: initial bolus of lactated Ringer solution before ERCP of 7.58mL/kg over 1h, lactated Ringer solution infusion during ERCP at 5mL/kg/h, post-ERCP bolus of 20mL/kg over 90 min	AH: 2443.5mL
			SH: lactated Ringer solution infusion at a rate of 1.5mL/kg/h at the start of ERCP. Fluids may be continued through the 90 min post-ERCP observation period	SH: 281.25 mL
Park et al	Korea	132/129	AH: intravenous lactated Ringer solution at a rate of 3.0mL/kg/h during the procedure, a bolus of 20mL/kg immediately after ERCP, followed by a post-ERCP rate of 3.0mL/kg/h for 8 h	AH: 3525 mL
			SH: intravenous lactated Ringer solution at a rate of 1.5mL/kg/h during ERCP and for 8h after ERCP without a bolus	SH: 1012.5 mL
M Alciva-Leon et al	Ecuador	326/326	AH: intravenous lactated Ringer solution at a rate of 3.0 mL/kg/h during ERCP, 20 mL/kg bolus after ERCP, and 3 mL/kg/h for 8 h after ERCP	AH: 3525mL
			SH: intravenous saline solution at a rate of 1.5 mL/kg/h during and for 8 h after ERCP	SH:1012.5 mL
Ramin et al	Iran	120/120	AH: a dose of 20 mL/kg/h of lactated ringer solution was given within 90 min before ERCP and 3 mL/kg /h was prescribed during ERCP, which lasted up to 8 hours. Then, it was reduced to 1.5 mL/kg /h if they did not have pain	AH:3525 mL
			SH: 1.5 mL/kg/h of lactated ringer solutionwas given during the ERCP, which lasted up to 8 h after ERCP.	SH:1012.5 mL

### 3.3 Risk of bias in included studies

**Figure 2:**
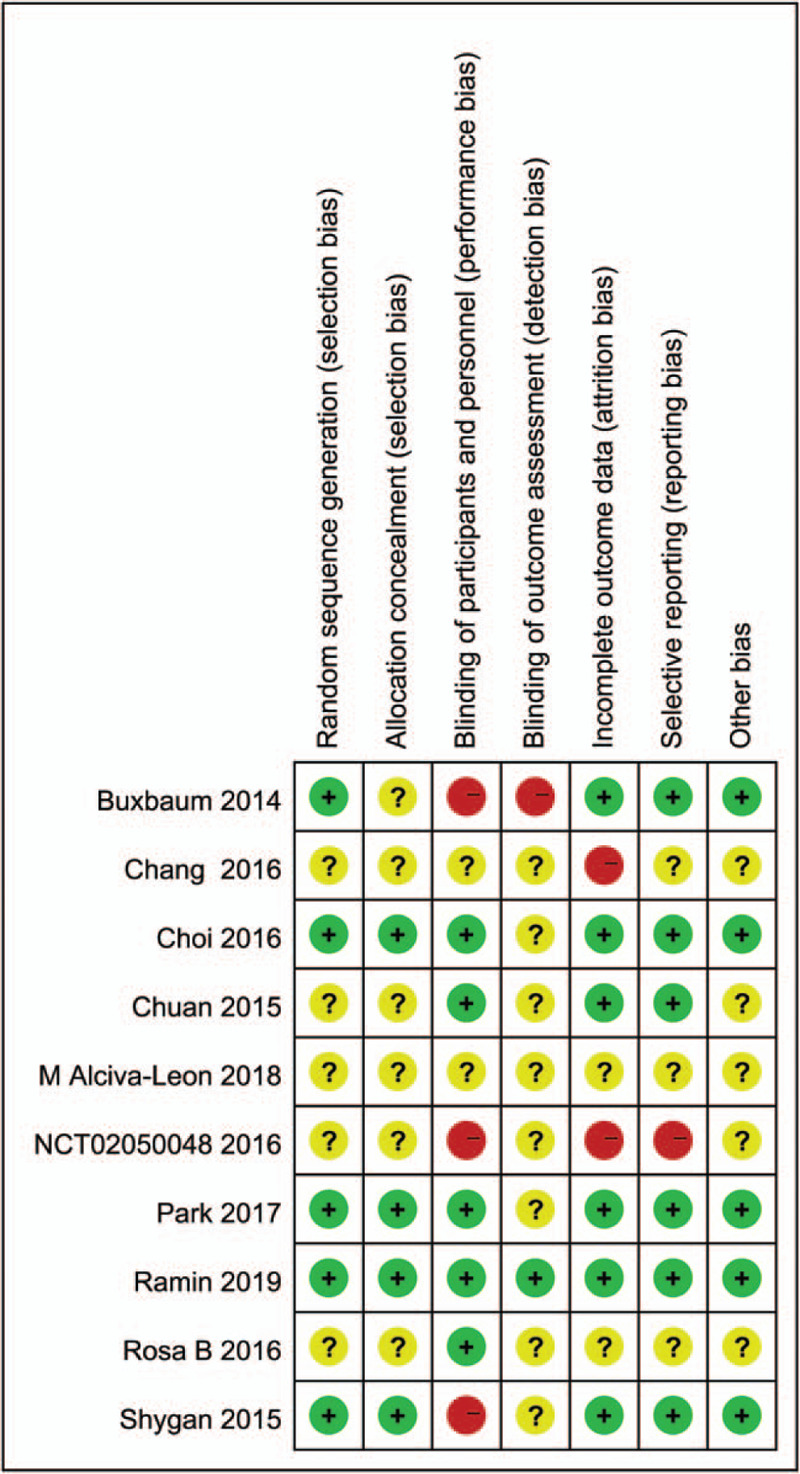
Risk of bias summary.

Among ten RCTs, 6 studies^[22–27]^ described detailed data in allocation sequence generation. 4 studies^[21,28–30]^ did not provide complete data about the method of the allocation sequence generation. Five studies^[21,22,28–30]^ did not provide enough information about allocation concealment method, while another five studies^[23–27]^ provided complete data in allocation concealment method. Four studies^[23,24,28,30]^ were double-blinded studies. 4 studies^[22,25–27]^ were single-blinded studies. The assessment of the risk of bias of included studies was illustrated in Figure 2.

### 3.4 Effects of interventions

#### 3.4.1 Incidence of PEP

This outcome was reported in ten studies^[21–30]^ with 2200 patients. A random effect model was used in our studies since the heterogeneity was detected. In this meta analyses, patients who received aggressive hydration had low risk of PEP as compared than the standard group (OR, 0.40; 95% CI, 0.26–0.63; *P* < .0001). Subgroup analysis was performed according to the time of initiation of aggressive hydration in different RCTs.

**Figure 3:**
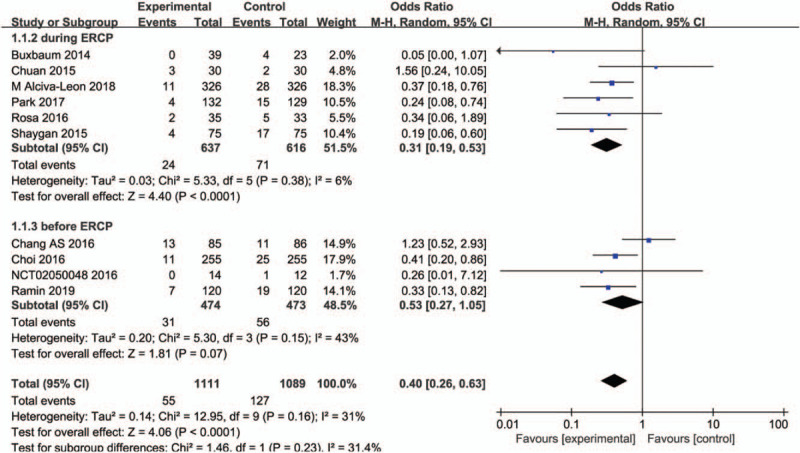
Forest plot of incidence of PEP between aggressive hydration and standard hydration.

In the first group^[21–25,27,28,30]^, the onset of fluid therapy was during the ERCP procedure, while in the second group,^[26,29]^ the initial bolus of lactated Ringer solution was before ERCP procedure. It was found that aggressive hydration had a significant effect on reducing the incidence of PEP (*I*^2^ = 6%; OR, 0.31; 95% CI, 0.19–0.53; *P* < .00001, random model) in the first group. However, there was no significant effect on reducing the incidence of PEP (*I*^2^ = 43%; OR, 0.53; 95% CI, 0.27–1.05; *P* = .07, random model) in the second group. The forest plot was shown in Figure 3. Sensitive analyses showed the conclusion would not change even if any study was omitted from the meta analyses.

#### 3.4.2 Incidence of Post-ERCP Hyperamylasemia

**Figure 4:**
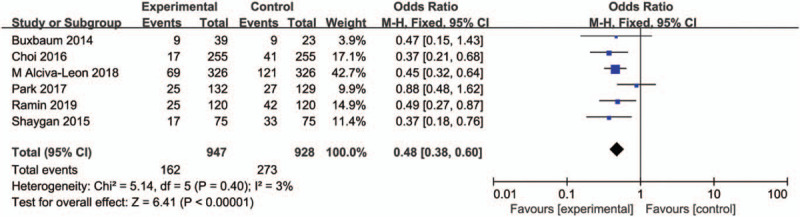
Forest plot of incidence of hyperamylasemia between aggressive hydration and standard hydration.

Six studies^[22–27]^ compared aggressive hydration with standard hydration in incidence of post-ERCP hyperamylasemia including 1875 patients. Heterogeneity was not detected among studies so we used a fixed effect model to assess the outcome (*I*^2^ = 3%). Meta analyses showed that patients who received aggressive hydration had low risk of post-ERCP hyperamylasemia than the standard group (OR, 0.48; 95% CI, 0.38–0.60; *P* < .0001). The forest plot was shown in Figure 4.

#### 3.4.3 Incidence of abdominal pain

**Figure 5:**
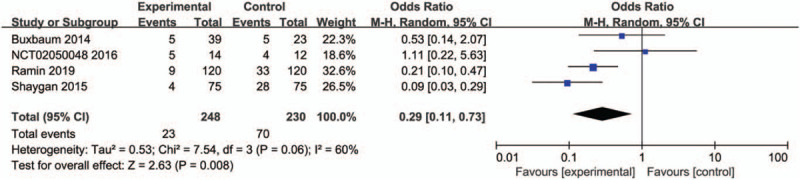
Forest plot of incidence of abdominal pain between aggressive hydration and standard hydration.

Four studies^[21–25,29,30]^ including 478 patients compared the incidence of abdominal pain between aggressive hydration and standard hydration during and after ERCP. Heterogeneity was detected in these studies so a random model was established to synthesize data by using Mantel Hansel method. Meta-analysis showed that there was significant difference between aggressive hydration and standard hydration (OR, 0.29; 95% CI, 0.11–0.73; *P* = .008). Figure 5 shows the forest plot. Sensitivity analysis detected that when the study by Ramin was removed, the results of the meta-analysis changed to no statistical significance (OR, 0.35; 95% CI, 0.08- 1.58; *P* = .17).

#### 3.4.4 Length of Stay of hospital (LoS)

**Figure 6:**
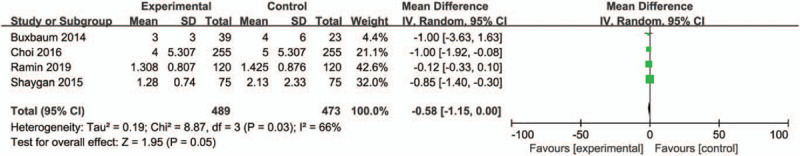
Forest plot of LoS between aggressive hydration and standard hydration.

Four studies compared aggressive hydration with standard hydration in LOS including 962 patients. Heterogeneity was detected among studies so we used a random effect model to assess the outcome. Meta analyses showed that the LoS of patients who received aggressive hydration was shorter than the standard group (mean difference = -0.58 day; 95% CI, -1.15 to-0.00; *P* = .05). The forest plot was shown in Figure 6.

#### 3.4.5 Incidence of fluid overload

**Figure 7:**
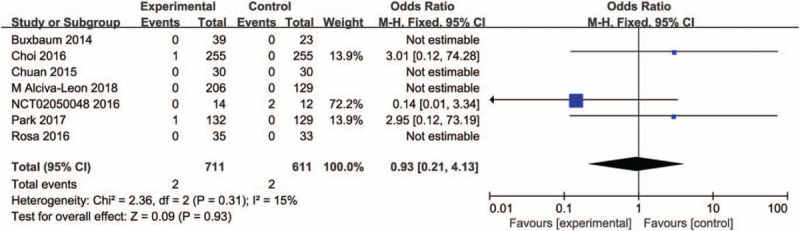
Forest plot of incidence of fluid overload between aggressive hydration and standard hydration.

Six studies compared aggressive hydration with standard hydration in incidence of fluid overload including 1322 patients. Heterogeneity was not detected among studies so we used a fixed effect model to assess the outcome. Meta analyses showed that there was no significance between the two groups (OR, 0.93; 95% CI, 0.21–4.13; *P* = .93). The forest plot was shown in Figure 7.

#### 3.4.6 Assessment of Quality of Evidence

Table 2 gives a grade assessment of the preventive effect of aggressive hydration on PEP. The quality of evidence for evaluating the preventive effect of Lactated Ringer solution on the aggressive hydration of PEP is moderate. The quality of evidence for evaluating the preventive effect of Lactated Ringer solution on hyperamylasemia after ERCP is high. The quality of evidence for evaluating the LoS of aggressive hydration of Lactated Ringer solution is low. The quality of evidence for evaluating the adverse effect of aggressive hydration of Lactated Ringer solution is very low.

**Table 2:**
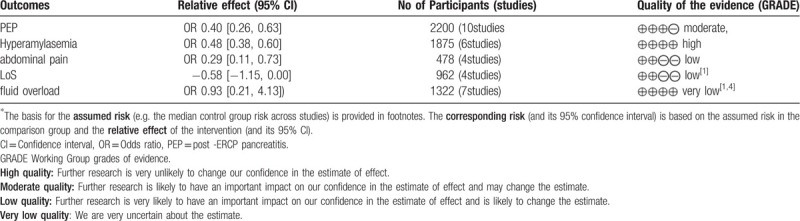
Assessment of quality of evidence.

## 4 Discussion

This mete analyses reviewed ten RCTs^[21–30]^ including 2200 patients. The results showed that patients who received aggressive hydration with Lactated Ringer Solution had low risk of PEP compared with those received standard hydration during or after the operation of ERCP. Aggressive hydration also decreased the incidence of Post-ERCP Hyperamylasemia and post-ERCP abdominal pain. Moreover, there was no significant difference in LOS and adverse events between the aggressive hydration group and the standard group. However, studies included in this meta analyses only used Lactated Ringer Solution, thus the conclusion could not apply to any other kind of liquid. Beside, total dosage of liquid therapy may matter.^[31]^ We calculated the fluid infusion doses during and 8 hours after ERCP for a 75 kg patient undergoing 1hour ERCP operation since we found that perioperative infusion of Lactated Ringer Solution differs from some RCTs to others. The onset of fluid therapy plays an extremely important role in influencing the incidence of PEP in our study. In summary, it is recommended to use aggressive hydration With Lactated Ringer Solution to prevent PEP.

We got the same results as the previous two published meta analysis^[19,20]^. More significant and large-scale studies were included in this study, the quality of the evidence is better than before, and the conclusions are relatively more credible. A new meta-analysis published in Pancreatology in 2019 came to the same conclusion,^[32]^ but the intervention in the study was aggressive hydration with lactate Ringer's solution or normal saline, while we only discussed aggressive hydration with lactate Ringer's solution. Considering the type of fluid that is actively hydrated, the amount of infusion, and the time of infusion may affect the outcome of fluid resuscitation, we performed a subgroup analysis, and the results showed that initiating active lactate Ringer's infusion during ERCP was statistically more significant in reducing the incidence of PEP. Enhanced perioperative fluid resuscitation can inhibit pancreatic inflammation by maintaining pancreatic microcirculation.^[31]^ Relative dehydration may aggravate microcirculation disturbance in ERCP patients. The indirect evidence supporting this theory is that preoperative elevation of blood urea nitrogen levels, as a measurement of hydration status, is associated with the development of PEP.^[33]^ Early massive infusion can replenish blood circulation in time, avoid insufficient blood circulation caused by fasting water before ERCP, thus ensuring pancreatic blood perfusion; sufficient pancreatic blood perfusion reduces pancreatic ischemia and hypoxia, reduces pancreatic cell function damage and pancreatic enzyme activation, prevents calcium ions from entering pancreatic cells, and interrupts cascade waterfalls caused by inflammatory factors. The cloth effect decreases the serum amylase level and the occurrence of PEP after ERCP.^[17,34]^ Besides, compared with normal saline, Lactated Ringer Solution can reduce the incidence of systemic inflammatory response syndrome and metabolic acidosis.^[35]^ Lactated Ringer solution can also stimulate anti-allergic reactions.

Previous prevention of PEP was mainly concentrated in the rectum, non-steroidal anti-inflammatory drugs and pancreatic stent implantation were recommended.^[15,16]^ In recent years, the prevention of PEP by Lactated Ringer Solution during perioperative period has been paid more and more attention.^[36]^ In addition to Lactated Ringer Solution, Acetate Ringer Solution and Carbonated Ringer Solution have been used in fluid therapy. However, there are few studies on Acetate Ringer Solution and Carbonated Ringer Solution, so this paper mainly discusses the preventive effect of aggressive hydration of Lactated Ringer Solution on PEP. Lactated Ringer Solution is widely available, safe, inexpensive and not easy to be damaged. Infusion of Lactated Ringer solution is a simple and convenient method to prevent PEP, which can be used as a supplement to prevent PEP at least in clinical practice.^[37]^

However, this paper also has some shortcomings and limitations. First of all, there are too few articles, only five are full texts. There are few clinical studies on PEP because of the limited number of participants and the ineffectiveness of potential therapies, which can be demonstrated by small studies. Secondly, the sample size is small and patients over 80 years old are excluded, but these patients may have ERCP. Thirdly, it is difficult to carry out effective research further because ERCP patients have heterogeneity in the etiology, pathological involvement and clinical stages of individual diseases. Therefore, in many cases, we have to make suggestions on weak evidence with moderate to high uncertainty. Fourthly, some of the results are incomplete and the description of adverse reactions is not detailed enough. Fifth, only hospitalized patients are allowed to register for an eight-hour infusion plan and monitor fluid overload closely. In many countries, most ERCP patients are in outpatient clinics, and outpatient treatment is unlikely to last for eight hours. Sixth, the quality of evidence is not high.

In conclusion, aggressive hydration with Lactated Ringer Solution during perioperative period of ERCP can prevent PEP. However, this conclusion still needs further evaluation. More randomized clinical trials are needed to support these findings in the future.

## Acknowledgments

We thank our collaborators who helped with the study.

## Author contributions

**Conceptualization:** Mengmeng Wu,Xiaoguang Lu.

**Data curation:** Mengmeng Wu, Shuaiyu Jiang.

**Formal analysis:** Mengmeng Wu, Yilong Zhong.

**Resources:** Yi Song, Zhiwei Fan.

**Software:** Yilong Zhong.

**Supervision:** Xiaoguang Lu, Xin Kang.

**Writing – original draft:** Mengmeng Wu.

**Writing – review & editing:** Mengmeng Wu.
